# Education‐Based Inequality in Edentulism and Functional Dentition Among Older Brazilian Adults: A Study Covering a Period of 20 Years

**DOI:** 10.1155/ijod/9983568

**Published:** 2026-01-09

**Authors:** Maria Luíza Viana Fonseca, Viviane Elisângela Gomes, Líria Sheila Chamane, Carlos Antonio Gomes da Cruz, Maria Luíza do Nascimento Silva, Ana Luíza Guerra Francisco, Loliza Luiz Figueiredo Houri Chalub, Raquel Conceição Ferreira

**Affiliations:** ^1^ Department of Community and Preventive Dentistry, School of Dentistry, Universidade Federal de Minas Gerais, Av. Presidente Antonio Carlos, 6627, Belo Horizonte, 31270-901, Minas Gerais, Brazil, ufmg.br

**Keywords:** dental health services, health inequality indicators, health inequality monitoring, health services for the aged, oral health, tooth loss

## Abstract

**Objective:**

To estimate the prevalence of edentulism and functional dentition (FD) in Brazil and assess the magnitude of inequalities among older adults, according to education in 2003, 2010, and 2023.

**Methods:**

This study used repeated cross‐sectional data from individuals aged 65–74 years who participated in the national oral health surveys (SB Brasil) conducted in 2003, 2010, and 2023. Edentulism was defined as the loss of all permanent teeth, and FD as the presence of 21 or more teeth. Educational level was categorized as: 0 (no schooling), 1–4, 5–8, 9–11, and ≥ 12 years of study. Absolute and relative inequalities in edentulism and FD were assessed using the slope index of inequality (SII) and the relative index of inequality (RII) based on education. Generalized linear models (GLMs) were applied with a logarithmic link function to estimate RII and an identity link function to estimate SII, adjusting for sex and age. The concentration index (CI) was calculated as twice the area between the concentration curve and the line of equality. All analyses accounted for the complex sampling design and sample weights.

**Results:**

The study included 5347 individuals in 2003, 7619 in 2010, and 9745 in 2023. The prevalence of edentulism was 53.33% in 2003 and 53.38% in 2010. In 2023, the prevalence significantly declined to 36.47%. FD prevalence was significantly higher in 2023 (23.94%) compared with 2003 (9.89%) and 2010 (11.45%). A worse oral health status was observed among individuals with lower educational levels. Indicators of absolute and relative inequality showed a significant increase in disparities, confirmed by the CI.

**Conclusion:**

There was an increase in inequalities in edentulism and FD, indicating that the reduction in tooth loss was greater among socioeconomically advantaged groups.

## 1. Introduction

Tooth loss is the most severe outcome of untreated dental caries and periodontal disease [1], and continues to be a major global public health issue. Tooth loss reflects access to dental care and often results from mutilating treatments with limited conservative options [2, 3]. Tooth loss negatively affects physical and mental health, self‐esteem, social relationships, employability, and overall quality of life [4, 5], and can be assessed by means of edentulism or by the number of remaining teeth. In response to its high prevalence, the World Health Organization (WHO) defined functional dentition (FD)—at least 21 natural teeth without prosthetics—as an oral health indicator [6].

Persistent disparities in tooth loss and FD are well documented [7–10], making it a global priority to reduce the rates of their occurrence. The Bangkok Declaration underscored the WHO’s concern regarding the social gradient in unmet oral health needs and called for intensified efforts to alleviate the associated burden, particularly among vulnerable groups [11]. Despite advances, many populations still face barriers to disease prevention and dental care, underscoring the need for universal access as a key public health policy priority [11]. In Brazil, the National Oral Health Policy (PNSB) has adopted a universal coverage model, with the aim of expanding access and reducing inequities through a health surveillance model that emphasizes continuous assessment. The purpose of these assessments would be to inform those responsible for elaborating public policies [12, 13]. Monitoring oral health disparities is therefore critical for understanding disease distribution, evaluating policies, and guiding interventions [14].

In this study, educational inequities in edentulism and FD among older Brazilian adults were analyzed, using data obtained from national surveys conducted in 2003, 2010, and 2023, spanning the PNSB implementation period. Analyzing older adults captures lifetime oral health impacts and potential cohort effects, while FD is a complementary outcome, offering sensitivity to changes in the severity of tooth loss.

Education‐based inequalities reflect social gradients linked to health knowledge, behaviors, resources, and service use. Individuals with higher education levels are more likely to engage in preventive behaviors, understand healthcare guidance, and make informed decisions [15–17]. Education enhances social standing, employment opportunities, and navigation of the healthcare systems, while also capturing the long‐term impact of early‐life circumstances on health outcomes [16, 17]—particularly relevant for disparities observed among older adults. Thus, the aim of this study was to estimate the prevalence of edentulism and FD in Brazil and to compare the educational inequalities in these outcomes among older Brazilian adults over two decades.

## 2. Methods

### 2.1. Design and Data Source

This study was based on repeated cross‐sectional analyses using secondary data from three national oral health surveys (SB Brasil) conducted in 2003, 2010, and 2023. The target population comprised older adults aged 65–74 years. Each survey accounted for variations in domains, strategies, and sample sizes, ensuring nationally and regionally representative estimates across Brazil’s five macro‐regions [18].

### 2.2. Population and Sampling

In 2003, sampling domains were the five regions and municipalities, with three‐ or four‐stage sampling depending on municipality size and age group. Fifty municipalities per region served as primary sampling units. Sample sizes for 65–74 years were based on 1986 dental caries data, ensuring 20% precision. In 2010, the frame included 32 domains (27 state capitals and 5 interior regions), using two‐ or three‐stage cluster sampling. Thirty primary sampling units (census tracts or municipalities) were selected per domain, with the sampling design aimed at achieving a coefficient of variation below 15% for estimates exceeding 10%. In 2023, 53 domains (26 capitals and 27 states) were surveyed. A two‐stage sampling method was used, selecting census tracts and households, targeting 400 participants per state for acceptable precision. Probability proportional to size was used, based on the private households. Detailed sampling procedures are available elsewhere [18–21] and in the appendix (Table S1).

### 2.3. Data Collection

Data were collected through household interviews and clinical oral examinations conducted under natural light, using WHO‐standardized dental mirrors and probes. In 2003 and 2010, each field team comprised an examiner and a recorder, while in 2023, an enumerator was added. Training in 2003 and 2010 involved 32 h of theoretical and practical activities [21]. In 2023, theoretical content was delivered through manuals and Moodle videos, and photographic simulations were used for examiner calibration [22, 23]. Examiner reliability was assessed using weighted or simple Kappa statistics (minimum thresholds: 0.65 in 2003/2010 [21]; 0.61 in 2023 [22]). Details on training and calibration procedures are provided in the appendix (Table S2).

### 2.4. Variables

Tooth loss was assessed according to WHO [24] criteria, based on the number of missing permanent teeth. Teeth lost due to caries or other reasons were included. Edentulism was defined as a loss of all 32 permanent teeth. FD was defined as the presence of ≥ 21 natural teeth [6].

Education was assessed as the number of years of study completed. Five categories were created: 0, 1–4, 5–8, 9–11, and ≥ 12 years of study, consistent with prior studies on oral health inequalities [9]. Covariates included sex (female and male) and age, categorized into 65–70 and 71–74 years.

### 2.5. Statistical Analysis

Datasets from the three surveys were harmonized, including a survey identifier. Weighted prevalence estimates for edentulism and FD were obtained overall and stratified by education. Change in prevalence (Δ2023–2010, Δ2023–2003, and Δ2010–2003) was tested using Δ divided by its standard error and 95% confidence intervals, evaluated with the Stata lincom command [25]. The annual percent change (APC) was calculated using exponential regression of the logarithm of the percentages as a function of time, according to ln (*Yt*) = *α* + *βt*, where APC = (exp[*β*] − 1) × 100, with 95% confidence intervals obtained using the delta method (nlcom). The APC represents the average annual rate of change in prevalence over time, where negative values indicate a decrease and positive values indicate an increase.

Inequalities were assessed using the slope index of inequality (SII), the relative index of inequality (RII), and the concentration index (CI), adjusted for age and sex. SII and RII were estimated by means of generalized linear models (GLMs) with appropriate link functions: log (RII) and identity (SII). Individuals were ranked by educational level and a fractional ridit‐score was assigned based on the midpoint of their range in the cumulative distribution of the sample [26]. Crude and adjusted SII and RII were derived from the model: gYi = *α* + *β*ri + *ε*, where Yi is the outcome (edentulism or FD) and ri is the corresponding fractional rank (ridit‐score) for the individual. *β* is the coefficient of interest and represents the crude RII or SII [26, 27]. In SII, values of zero indicate no inequality; positive SII suggests higher prevalence among advantaged groups, negative values indicate the opposite. RII values of 1 indicate no inequality; values >1 show concentration among higher‐educated individuals; values <1 among lower‐educated groups [26]. Temporal trends in inequality were evaluated by adding interaction terms between the ridit‐score and survey year to the models [27]. A statistically significant coefficient for the interaction term implies changes in the adjusted RII and SII over time.

The CI quantifies socioeconomic inequalities in health outcomes based on the concentration curve, which plots the cumulative percentage of the outcome against the cumulative percentage of the population ranked by ascending educational level. The index is defined as twice the area between the concentration curve and the line of equality (the 45‐degree line). It is negative when the curve lies above the line of equality, indicating that the health outcome is disproportionately concentrated among the less educated, and a positive value when concentrated among the more educated. For adverse outcomes, such as edentulism, a negative CI reflects greater prevalence among disadvantaged groups [26]. Calculation of the CI and its standard error, were used to derive 95% confidence intervals, in accordance with the methodology described by O’Donnell et al. [28], using the conindex package in Stata. The *Z*‐test assessed equality of concentration indices across survey years.

Sampling weights were calculated as the inverse of the product of the sampling fractions for all surveys [19, 20, 29]. In 2023, to minimize selection and response bias in the survey, these weights were adjusted using post‐stratification weights through the Rake method. This procedure aimed to align the joint distributions of sex, age, and education between the sample and the reference population [19]. The weight variable was applied in all analyses. Variance estimation accounted for the clustering of individuals within primary sampling units and the stratification, using the svyset command in Stata, as detailed in appendix Methods 1. All prevalence estimates, confidence intervals and regression coefficients were computed using survey‐design procedures that take into account complex sampling. Survey weights and design adjustments were applied to minimize potential bias arising from item nonresponse. Analyses were based on complete cases and performed in Stata 18.0 (StataCorp LP, College Station, TX, USA).

### 2.6. Ethics

The three surveys were approved by the National Research Ethics Committee (2003: protocol 1356; 2010: protocol 15.498; and 2023: protocol 4.823.054). Participants provided Informed Consent. The present analysis was based on anonymized, publicly available data, and therefore, did not require additional ethical approval.

## 3. Results

The study included 5347 individuals aged 65–74 years in 2003, 7619 in 2010, and 9745 in 2023. Table S3 shows the number and percentage of complete records for each variable across the three surveys. The variable with the highest proportion of missing data was education, with completeness ranging from 97.40% in 2010 to 98.41% in 2003. When considering all variables simultaneously, the proportion of complete records was 97.22% (*n* = 22,080) for the total sample, 98.41% (*n* = 5262) in 2003, 96.02% (*n* = 7316) in 2010, and 97.51% (*n* = 9502) in 2023. Across all surveys, the majority of participants lived in the Southeast region, were female, and aged 65–70 years. The majority had 1–4 years of study; however, the proportion with no formal education declined from 28.98% in 2003 to 11.53% in 2023, while those with ≥ 12 years of study increased from 2.27% to 15.25% (Table 1).

**Table 1:** Characteristics of individuals aged 65–74 years participating in the three Brazilian oral health surveys conducted in 2003, 2010, and 2023.

Variables	Surveys		
	2003 (*n* = 5347)	2010 (*n* = 7619)	2023 (*n* = 9745)
	%w (95% CI)^a^	%w (95% CI)^a^	%w (95% CI)^a^
Regions			
North	5.07 (3.82; 6.68)	5.14 (4.06; 6.49)	5.63 (4.75; 6.65)
Northeast	26.95 (24.36; 29.71)	9.76 (8.78; 10.84)	24.04 (21.21; 27.12)
Southeast	45.89 (41.65; 50.18)	62.85 (59.53; 66.05)	46.95 (42.46; 51.48)
South	16.61 (15.05; 18.30)	16.18 (14.48; 18.05)	16.67 (14.22; 19.46)
Central‐west	5.48 (4.89; 6.14)	6.05 (5.32; 6.88)	6.71 (5.20; 8.61)
Sex			
Male	43.80 (40.87; 46.77)	39.58 (37.31; 41.9)	40.26 (38.74; 41.81)
Female	56.20 (53.23; 59.13)	60.42 (58.10; 62.69)	59.74 (58.19; 61.26)
Age group (completed years)			
65–70	65.21 (59.36; 70.63)	63.35 (60.41; 66.19)	65.21 (61.86; 68.41)
71–74	34.79 (29.3; 40.64)	36.65 (33.81; 39.59)	34.79 (31.59; 38.14)
Education (years of study)			
0	28.98 (26.17; 31.97)	14.8 (12.27; 17.75)	11.53 (9.80; 13.51)
1–4	49.12 (44.42; 53.82)	49.05 (45.29; 52.83)	27.88 (23.98; 32.14)
5–8	13.64 (11.97; 15.50)	20.43 (18.00; 23.09)	26.24 (23.68; 28.97)
9–11	5.98 (5.10; 6.99)	8.94 (7.46; 10.68)	19.10 (16.82; 21.62)
≥12	2.28 (1.72; 2.9)	6.78 (5.11; 8.94)	15.25 (12.92; 17.91)

^a^Analyses accounted for the effects of sample design and weighting.

**Figure 1:**
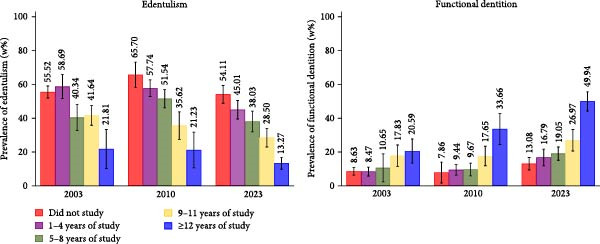
Prevalence of edentulism and functional dentition according to educational level among individuals aged 65–74 years participating in the three national oral health surveys in Brazil in 2003, 2010, and 2023. Analyses accounted for the effects of sample design and weighting. w% = weighted prevalence.

**Figure 2:**
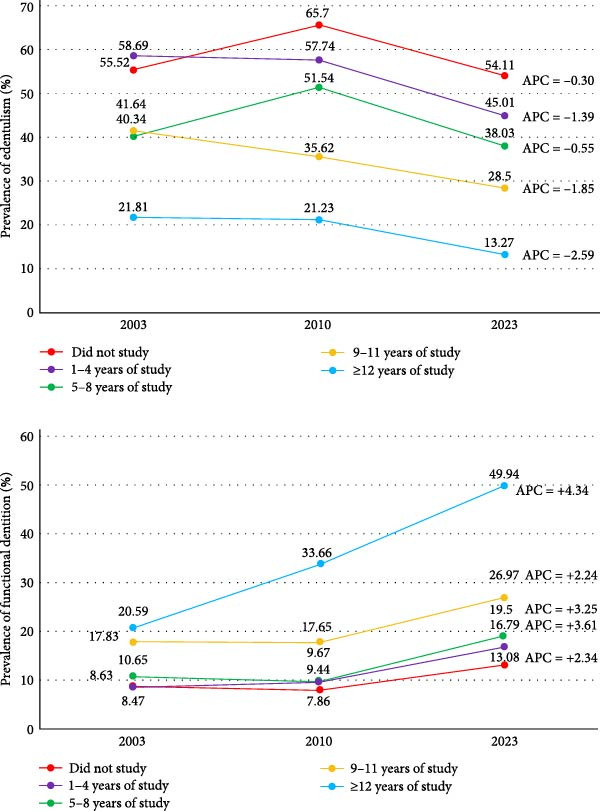
Annual percent change (APC) from 2003 to 2023 in the prevalence of edentulism and functional dentition among individuals aged 65–74 years, by education level, Brazil.

There was no significant change in the prevalence of edentulism between 2003 (53.34%; 95% CI: 49.58–57.09) and 2010 (53.38%; 95% CI: 49.73–57.03). However, a significant decline was observed in 2023 (36.48%; 95% CI: 33.37–39.59) compared to 2003 and 2010 (Figure 1 and Table S4). The estimated APC over the study period was −2.01% (95% CI: −3.44; −0.57) (Figure 2 and Table S4).

For FD, no significant difference was observed between 2003 (9.89%; 95% CI: 8.41–11.37) and 2010 (11.45%; 95% CI: 9.22–13.68), but prevalence increased significantly in 2023 (23.95%; 95% CI: 21.24–26.65) (Figure 1 and Table S4). The estimated APC over the study period was of +4.69% (95% CI: + 2.81; + 6.56) (Figure 2 and Table S4).

In all years, edentulism was found more frequently among individuals with lower education, showing a social gradient, particularly in 2010 and 2023. The prevalence of FD was consistently higher among those with higher education levels (Figure 1). The APC indicated that the reduction in edentulism over the study period was most pronounced among individuals with 9–11 years of education (APC = –1.85%) and those with ≥ 12 years (APC = −2.59%) (Figure 2 and Table S3). For FD, the increase was more pronounced among individuals with the highest education (APC = + 4.34%) (Figure 2 and Table S4). Detailed stratified analyses are shown in appendix Table S4.

Inequality measures indicated that edentulism continued to be concentrated among the least educated, with negative SII values and RII < 1 across all years (Table 2). Absolute differences in the prevalence of edentulism between the highest and lowest educational levels were approximately 16, 37, and 42 percentage points in 2003, 2010, and 2023, respectively. Relative disparities decreased by approximately 25%, 48%, and 70% across the same periods (Table S4). Although edentulism declined overall, inequality increased, notably in 2023. For FD, positive SII and RII >1 indicated concentration among the most educated, with increasing inequality over time (Table 2). Absolute differences in the prevalence of FD were approximately 6, 15, and 31 percentage points in 2003, 2010, and 2023, respectively. Prevalence was 1.76, 3.81, and 4.88 times higher among the most educated compared with those with no education (Table S4).

**Table 2:** Slope index of inequality (SII), relative index of inequality (RII), and absolute concentration index, with 95% confidence intervals (CI), for education‐based inequalities in edentulism and functional dentition among Brazilian older adults in 2003 (*n* = 5262), 2010 (*n* = 7316), and 2023 (*n* = 9502) with *p*‐values for trends in inequality magnitude over time.

Surveys	Edentulism	Functional dentition
	Adjusted SII^1^ (95% CI)	Adjusted SII^1^ (95% CI)
2003	−0.1644 (−0.2269; −0.1018)^a^	0.0601 (0.2402; 0.9621)^a^
2010	−0.3650 (−0.4538; −0. 2762)^bc^	0.1452 (0.0846; 0.2059)^a^
2023	−0.4184 (−0.4866; −0.3503)^c^	0.3086 (0.2368; 0.3803)^b^
Coefficient from two‐way interaction ridit‐score × survey	**−0.1151 (−0.1619; −0.0684)**	**0.1246 (0.8982; 0.1593)**
*p*‐Value for trend	**<0.001**	**<0.001**
	Adjusted RII^1^ (95% CI)	Adjusted RII^1^ (95% CI)
2003	0.7683 (0.6683; 0.8833)^a^	1.7556 (1.0666; 2.8894)^a^
2010	0.5234 (0.4430; 0.6183)^b^	3.8106 (1.7489; 8.3027)^ab^
2023	0.3349 (0.2772; 0.4044)^c^	4.8794 (3.2772; 7.2650)^b^
Coefficient from two‐way interaction ridit‐score × survey	**0.6738 (0.6086; 0.7460)**	**1.6104 (1.1179; 2.3198)**
*p*‐Value for trend	**<0.001**	**0.011**
	Concentration index (95% CI)	Concentration index (95% CI)
2003	−0.0530 (−0.0885; −0.0175)^a^	0.0981 (−0.0611; 0.2573)^a^
2010	−0.1015 (−0.1375; −0.0655)^b^	0.1968 (0.0730; 0.3207)^b^
2023	−0.1852 (−0.2220; −0.1485)^c^	0.2368 (0.1957; 0.2780)^c^
Differences of concentration index among surveys	***F*-stat: 16.91, *p*-value <0.001**	***F*-stat: 24.03, *p*-value <0.001**

*Note:* The RII and SII were adjusted for age and sex. Bold values indicate statistically significant trends (*p*  < 0.05). Different superscript lowercase letters indicate statistically significant differences resulting from the *F*‐test, comparing concentration index between two surveys.

^1^Different superscript lowercase letters in the vertical represent a significant interaction term between ridit‐score × survey, keeping only two surveys in the model at a time

For edentulism, SII decreased from 2003 to 2010 and no significant difference was observed between 2010 and 2023. RII was significantly lower in 2023 compared with 2003 and 2010, and lower in 2010 compared with 2003. For FD, SII showed no significant change between 2003 and 2010 but increased significantly from 2010 to 2023. There was no significant difference in RII between 2003 and 2010, and between 2010 and 2023, but it was significantly higher in 2023 compared with 2003.

**Figure 3:**
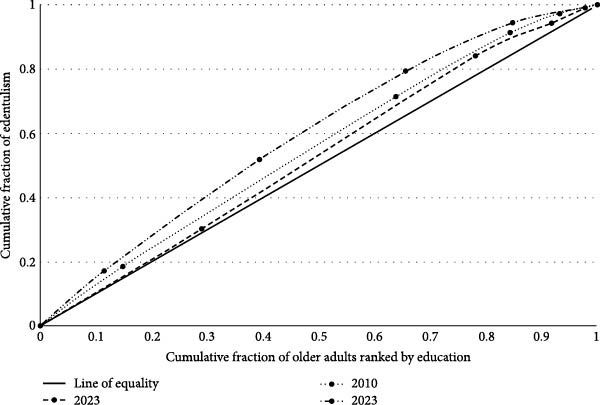
Relative concentration curves for edentulism in 2003, 2010, and 2023 Brazilian oral health surveys.

**Figure 4:**
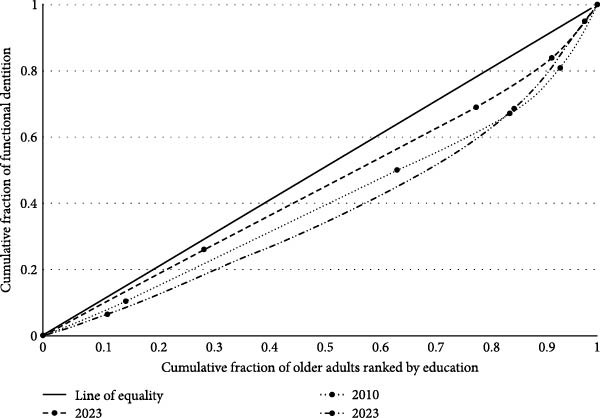
Relative concentration curves for functional dentition in 2003, 2010, and 2023.

CI values were consistently negative for edentulism and positive for FD, reflecting persistent educational inequalities. The CI increased significantly between 2003 and 2010, and again from 2010 to 2023 (Table 2). Edentulism curves were consistently above the equality line, with greater inequality in 2023. For FD, curves were below the equality line, indicating higher prevalence among the more educated (Figures 3 and 4).

## 4. Discussion

This study showed a decline in tooth loss among older Brazilian adults from 2003 to 2023. However, educational inequalities persist, and both absolute and relative disparities widened over time.

Our findings on the decrease in tooth loss aligned with global burden of disease (GBD) international trends [1, 2]. In Brazil, the National Health Survey also showed a 4.70 percentage‐point reduction in edentulism among adults aged 60+ between 2013 and 2019 [30]. Improvements reflect broader exposure to fluoride, changes in dental care practices moving toward prevention and conservation, technological advances and expanded access to dental services [31, 32]. In Brazil, the PNSB marked a shift from a curative to a preventive care model—particularly after the 2004 PNSB which most likely played a major role in reducing tooth loss among older adults. This reorientation expanded preventive and conservative practices [12, 13] overcome the historical dominance of mutilative treatments and limited access to care. Empirical data supported this transition, showing a steady decline in tooth extractions performed by the unified health system (SUS) between 1998 and 2012 [33] and sustained decreases in extraction rates through 2018 [34]. Moreover, improvements observed among today’s older adults are unlikely to have resulted from fluoride exposure during childhood, as those aged 65–74 in 2023 were born before the widespread adoption of water fluoridation and fluoride toothpaste [35]. Despite improvements, edentulism continues to show higher rates in Brazil than the global average (22.70%). Additionally, population aging [36]—from 14.5 million older adults in 2003 to 32.1 million in 2023—means that the absolute number of edentulous individuals has grown, consistent with GBD projections [1].

Educational disparities persisted, with better outcomes among more educated individuals, as previously reported [7–10]. Among the most educated, prevalence approached or met the global 2030 targets. These findings reflect material and behavioral pathways. From a material perspective, education enhances access to income, employment [16, 37], and resources (e.g., ability to afford a healthy diet and access to health and public services) that promote health and protect from exposure to risk factors that are detrimental to health (e.g., poor housing conditions, pollution, hazardous work environments) [37]. According to the cultural/behavioral pathway, education enhances the development of knowledge and skills that contribute to self‐care practices, such as proper toothbrushing, healthy eating, and regular use of health services [16, 17]. Education also broadens individuals’ understanding of their health needs, risks, and preventive measures, and positively influences social participation, support, and cohesion‐factors that are recognized as being associated with better general and oral health [16, 17]. These findings highlight education as a key socioeconomic marker shaping health behaviors. Therefore, structural interventions targeting underlying social and economic conditions are essential for sustainable outcomes, recognizing that health behaviors are shaped by complex social contexts [14, 38].

A life‐course perspective helps explain persistent inequalities. Individuals with lower education accumulate disadvantages through a range of material, social, psychological and biological advantages and disadvantages, and social experiences throughout life [38]. Evidence shows that childhood socioeconomic conditions have a lasting impact on adult edentulism [7, 39]. Qualitative studies have shown that older Brazilian adults link socioeconomic status (SES) to lifelong barriers in oral care, viewing tooth loss as inevitable [40]. Historically, dental care was mainly pain‐driven, with extractions being the primary accessible treatment until 2004, when the SUS shifted away from a mutilative care model [3].

Widening inequalities seem to be driven by unequal improvements over time. Between 2003 and 2010, edentulism increased among individuals with no education but declined among those with higher education. This pattern may reflect the expansion of public oral health services following the implementation of the 2004 PNSB [12], with improved access to care for previously excluded groups. Conversely, better‐educated individuals maintained healthier trajectories. As noted by Tsakos et al. [14], those with better initial oral health tend to accumulate lifelong advantages. Although edentulism declined across all educational levels between 2023 and 2003, the pace of reduction varied from 2.54% among those with no education to 39.16% among individuals with 12 or more years of education. Between 2010 and 2023, there were no significant changes in absolute inequalities, indicating persistent gaps between educational groups, while relative inequalities increased. This was driven by a proportionally greater decline among the more educated (around 40%) compared with those with lower education (17.64%), meaning improvements were faster among the better educated, impacting relative disparity measures. A similar disproportionate reduction was observed in the APC analysis, showing a significant annual decline of 2.59% in the prevalence of edentulism among the most educated, compared with a nonsignificant decline of 0.30% among those with the lowest education levels. These patterns highlight the influence of structural determinants, as socially advantaged groups tend to benefit more quickly and extensively from health improvements. This aligns with the theory of fundamental causes [41], which states that access to resources such as education allows privileged groups to improve leverage of health innovations, and with the inverse equity hypothesis [42], which suggests that new health interventions initially widen inequalities by benefiting higher SES groups first, only later reducing gaps as access becomes widespread.

For FD, the observed increase in absolute and relative inequalities between 2003 and 2010 also appears to have been driven by disproportionately greater improvements among older adults with higher education, indicating that progress primarily benefited socioeconomically advantaged groups. All relative disparity measures (SII, RII, and CI) indicated greater inequality in FD than in edentulism, suggesting that FD is more sensitive to recent oral health care improvements, such as preventive interventions and conservative treatments. These services are more readily accessed by individuals with higher education. However, after 2010, gains were also seen among less educated groups, most likely due to expanded public oral health services after implementing the PNSB in 2004 and the creation of centers for dental specialties from 2006, contributing to tooth preservation through treatments such as endodontics [12]. SUS data show a reduction in extractions [33, 34] and greater use of public dental services by socially disadvantaged groups using public services in 2019, compared with much lower rates among higher‐SES groups [43]. Nevertheless, differences persist, as individuals with higher education are in a better position to engage in behavior‐change interventions. Although service use only partially explains inequalities in edentulism [9], natural teeth number [44], and FD [10], it is crucial to explore how these inequalities vary across socioeconomic strata and according to the type of services used (public vs. private). The continued expansion of access to care, guided by the principles of universality and equity, should remain a central goal of the PNSB.

In summary, while notable advances in oral health among older Brazilians have been achieved, widening educational disparities underscore the importance of strategies that address broader structural determinants of health. Our findings are consistent with studies conducted in low‐ and middle‐income countries (LMICs), which have demonstrated the existence of inequalities in tooth loss and dental caries, the main cause of tooth loss, associated with socioeconomic indicators such as income and education. The poorest outcomes were consistently observed among individuals belonging to groups with lower SES [45–47]. Moreover, they underscore the importance of monitoring oral health inequalities by strengthening health surveillance systems and ensuring their use to guide PNSB actions focused on health planning and resource allocation, intending to benefit the most vulnerable groups more equitably.

### 4.1. Strengths and Limitations

This study used three national surveys to analyze trends in tooth loss among the older adult population. The results showed consistent patterns across the employed complementary analytical approaches for comparing education‐based inequalities over time (Δ, APC, and inequality indices), revealing the robustness of our findings. WHO‐standardized diagnostic criteria, rigorous training, and calibration ensured consistent comparisons, thus strengthening validity. Large sample sizes supported precise estimates. However, different survey periods require contextual interpretation, as changes in social, economic, and policy environments may have influenced oral health indicators, and its cross‐sectional design prevents causal inference. Complex inequality measures (SII, RII, and CI) were used, incorporating full socioeconomic distributions and allowing standardized comparisons. SII and RII, regression‐based, are particularly recommended for monitoring inequalities [26]. Nonetheless, education alone does not capture the full eco‐social framework of determinants [15], and education measured in older age may underestimate early‐life inequalities. Education can not reflect other early‐life disadvantages (such as poor living conditions, limited access to care, malnutrition, or parental education). Moreover, some individuals may have improved their education later in life, but the effects of early disadvantage (like poor oral health or limited access to preventive care in childhood) remain.

## 5. Conclusion

Between 2003 and 2023, the prevalence of edentulism declined and FD increased among older Brazilian adults. However, both absolute and relative inequalities widened, indicating that improvements were concentrated among socioeconomically advantaged groups.

## Disclosure

The funding agencies had no role in the design, conduct, analysis, or reporting of this study.

## Conflicts of Interest

The authors declare no conflicts of interest.

## Author Contributions

**Maria Luíza Viana Fonseca:** formal analysis, data curation, visualization, writing – original draft, writing – review and editing. **Viviane Elisângela Gomes:** writing – original draft, writing – review and editing, visualization. **Líria Sheila Chamane:** formal analysis, writing – original draft, writing – review and editing. **Carlos Antonio Gomes da Cruz, Maria Luíza do Nascimento Silva, and Ana Luíza Guerra Francisco:** writing – original draft, writing – review and editing. **Raquel Conceição Ferreira:** conceptualization, supervision, methodology, formal analysis, data curation, writing – original draft, writing – review and editing, supervision.

## Funding

This work was supported by the Fundação de Amparo à Pesquisa do Estado de Minas Gerais, FAPEMIG, Brazil (Grant APQ‐00763‐20), the Coordenação de Aperfeiçoamento de Pessoal de Nível Superior, CAPES, Brazil (Grant 88887.004344/2024‐00), and the Conselho Nacional de Desenvolvimento Científico e Tecnológico, CNPq, Brazil (Grant 310938/2022‐8). This work was carried out with the support of the Coordenação de Aperfeiçoamento de Pessoal de Nível Superior (CAPES) ‐ Funding Code 001.

## Supporting Information

Additional supporting information can be found online in the Supporting Information section.

## Supporting information


**Supporting Information** Table S1. Sampling procedures used in the SB Brasil Surveys (2003, 2010, and 2023), including sampling domains, selection stages, primary sampling units, sample size calculations, and methodological notes. Table S2. Examiner training and calibration procedures for the SB Brasil Surveys (2003, 2010, and 2023), with details on training approaches, calibration methods, and minimum acceptable Kappa values. Methods 1: detailed statistical methods used in the analyses, including weighting, stratification, calculation of changes in prevalence (Δ), and Stata code examples. Table S3. Number and percentage of complete records for each analyzed variable by survey. Table S4. Prevalence of edentulism and functional dentition by education level among older Brazilian adults in 2003, 2010, and 2023, with differences between surveys (∆2010–2003, ∆2023–2003, and ∆2023–2010).

## Data Availability

The databases are made available by the Ministry of Health of Brazil, upon the researcher’s request, through the link https://abre.ai/bancosdedadossbbrasil.
