# Supplementary material for: Education‐Based Inequality in Edentulism and Functional Dentition Among Older Brazilian Adults: A Study Covering a Period of 20 Years
Source: Int J Dent. 2026 Jan 9;2026:9983568. doi: 10.1155/ijod/9983568 (PMC12789184; doi:10.1155/ijod/9983568)
Supplement: Supplementary file 1 — Supporting Information Table S1. Sampling procedures used in the SB Brasil Surveys (2003, 2010, and 2023), including sampling domains, selection stages, primary sampling units, sample size calculations, and methodological notes. Table S2. Examiner training and calibration procedures for the SB Brasil Surveys (2003, 2010, and 2023), with details on training approaches, calibration methods, and minimum acceptable Kappa values. Methods 1: detailed statistical methods used in the analyses, including weighting, stratification, calculation of changes in prevalence (Δ), and Stata code examples. Table S3. Number and percentage of complete records for each analyzed variable by survey. Table S4. Prevalence of edentulism and functional dentition by education level among older Brazilian adults in 2003, 2010, and 2023, with differences between surveys (∆2010–2003, ∆2023–2003, and ∆2023–2010). [file IJOD-2026-9983568-s001.docx]

**Table S1. Sampling Procedures in the SB Brasil Surveys (2003, 2010, and 2023)**

| **Survey Year** | **Sampling Domains** | **Sampling Stages** | **Primary Sampling Units (PSU)** | **Sample Size Calculation** | **Notes** |
| --- | --- | --- | --- | --- | --- |
| **2003** | 5 macro-regions and selected municipalities | 3-4 stages (depending on municipality size and age group) | 50 municipalities per region | Based on the 1986 dental caries prevalence, 20% precision | Stratification by municipality size |
| For municipalities with up to 50,000 inhabitants and for the selection of 12-year-old adolescents, sampling was conducted in three stages (municipalities, schools, participants). In these municipalities, for the other age groups, sampling also occurred in three stages (municipalities, urban blocks or rural villages, and households). In municipalities with more than 50,000 inhabitants, in the capitals, sampling for 5-year-old children and 12-year-old adolescents was carried out in two stages (schools or daycare centers, participants), and for the other age groups, in three stages (census tracts, blocks, and households). In other municipalities with more than 50,000 inhabitants, sampling for the 5- and 12-year-old age groups occurred in three stages (municipalities, schools, participants), and for the remaining age groups, in four stages (municipalities, census tracts, blocks/rural villages, households). The primary sampling units were 50 municipalities in each of the five regions of the country, with 10 municipalities in each population size stratum, totaling 250 municipalities, and 20 schools in each municipality for the 12-year-old adolescents. The sample size for the 65–74 age group was calculated for each macro-region based on caries prevalence estimates from 1986. Values were determined considering a 20% precision level and adjusted for finite populations.^17^. | | | | | |
| **2010** | 27 capitals and 5 interior regions | 2-3 stages (depending on domain) | 30 census tracts or municipalities per domain | Designed for <15% CV for prevalence >10% | Inclusion of state capitals |
| The sampling plan included 32 geographic domains: 27 corresponding to state capitals and five domains representing non-capital municipalities in each of the five macro-regions. A cluster sampling design was used, structured in two stages for the capitals and municipalities (census tract and household) and in three stages for non-capital areas (municipality, census tract, and household). In each domain, 30 Primary Sampling Units were selected—30 census tracts in the capitals and 30 municipalities in non-capital areas. The Secondary Sampling Units were households, and the required number of households for the sample was determined based on the average number of individuals from each age group per household. The sample size calculation for the 65–74-year age group was based on the minimum precision required for prevalence estimates, accepting coefficients of variation <15% for prevalences above 10%. The sample size was defined as 250 individuals per domain, totaling 8,000 individuals aged 65–74 years. | | | | | |
| **2023** | 26 capitals + 27 states (total 53 domains) | 1-2 stages (census tract → household) | Census tracts | 330 - 400 participants per state for the purpose of obtaining estimates with acceptable precision | State was a study domain |
| In 2023, the study domains consisted of the 26 state capitals and the 27 states (Federative Units) of Brazil, totaling 53 geographic domains. Sampling was conducted in one (5 and 12 years old) or two stages (15-19, 35-44, and 65-74 age groups): the primary sampling unit was the census tract, and the secondary unit was the household. For the 65–74-year-old age group, the sample size was set at 300 individuals per capital city. This was supplemented by a sample of 100 individuals from census tracts in non-capital areas of each state, resulting in a total of 400 individuals per unit. This design enabled the estimation of prevalence and mean values of key oral health outcomes with acceptable precision. Sampling units were selected using probability proportional to size, based on the number of permanent private households. | | | | | |

**Table S2. Supplementary Table 2. Examiner Training and Calibration Procedures**

| **Survey Year** | **Training Approach** | **Calibration Method** | **Minimum Acceptable Kappa** | **Notes** |
| --- | --- | --- | --- | --- |
| 2003 | 32 hours (theoretical + practical) | Field calibration with observed exams | 0.65 | Field-based sessions |
| 2010 | 32 hours (theoretical + practical) | Field calibration with observed exams | 0.65 | Consistent with 2003 |
| 2023 | Theoretical (manuals + online videos for a minimum of 16 hours); Practical (photographic simulation) | *In lux* photo simulations | 0.61 | Use of technology for training |

**Supplementary Methods 1. Detailed Statistical Methods**

*Weighted Prevalence Estimation*

- Applied survey weights are specific to each survey year.
- Stratified estimates by educational attainment groups.
- Confidence intervals obtained using Taylor linearization.

*Change in Prevalence Over Time*

- Δ = Difference in prevalence between years.
- Standard error (SE) is calculated based on variance estimates for complex surveys.
- 95% Confidence Interval (CI) = Δ ± 1.96 × SE.

| Examples of Stata codes:  *--------------------------------------------------------*  * Set sample design  *--------------------------------------------------------*  svyset sector [pweight = wgt], strata(single extract) vce(linearized) single unit(certainty)  *--------------------------------------------------------*  * Mean estimation of edentulism for each survey year  *--------------------------------------------------------*  svy: mean edent_perc, over(survey)  * Pairwise comparisons between survey years  lincom [c.edent_perc@3.survey] - [c.edent_perc@2.survey]  lincom [c.edent_perc@3.survey] - [c.edent_perc@1.survey]  lincom [c.edent_perc@2.survey] - [c.edent_perc@1.survey]  *--------------------------------------------------------*  * Mean estimation of edentulism by educational level  *--------------------------------------------------------*  * No education  svy, subpop(escol1): mean edent_perc, over(survey)  lincom [c.edent_perc@3.survey] - [c.edent_perc@2.survey]  lincom [c.edent_perc@3.survey] - [c.edent_perc@1.survey]  lincom [c.edent_perc@2.survey] - [c.edent_perc@1.survey]  * 1 to 4 years of schooling  svy, subpop(escol2): mean edent_perc, over(survey)  lincom [c.edent_perc@3.survey] - [c.edent_perc@2.survey]  lincom [c.edent_perc@3.survey] - [c.edent_perc@1.survey]  lincom [c.edent_perc@2.survey] - [c.edent_perc@1.survey]  * 5 to 8 years of schooling  svy, subpop(escol3): mean edent_perc, over(survey)  lincom [c.edent_perc@3.survey] - [c.edent_perc@2.survey]  lincom [c.edent_perc@3.survey] - [c.edent_perc@1.survey]  lincom [c.edent_perc@2.survey] - [c.edent_perc@1.survey]  * 9 to 11 years of schooling  svy, subpop(escol4): mean edent_perc, over(survey)  lincom [c.edent_perc@3.survey] - [c.edent_perc@2.survey]  lincom [c.edent_perc@3.survey] - [c.edent_perc@1.survey]  lincom [c.edent_perc@2.survey] - [c.edent_perc@1.survey]  * 12 or more years of schooling  svy, subpop(escol5): mean edent_perc, over(survey)  lincom [c.edent_perc@3.survey] - [c.edent_perc@2.survey]  lincom [c.edent_perc@3.survey] - [c.edent_perc@1.survey]  lincom [c.edent_perc@2.survey] - [c.edent_perc@1.survey] |
| --- |

*Measurement of Inequalities*

Slope Index of Inequality (SII)

- Regression model with identity link:
- Coefficient β of the model = Absolute difference in outcome prevalence between the lowest and highest educational groups.

Relative Index of Inequality (RII)

- Regression model with log link:
- Coefficient β of the model = Prevalence ratio comparing the highest to the lowest educational groups.

Temporal Trends in Inequalities

- Interaction terms between ridit score and survey year were included in GLMs.
- Statistical significance of interaction terms is assessed to infer changes in inequalities over time.

| Examples of Stata codes:  *--------------------------------------------------------*  * Relative Index of Inequality (RII) – single survey  *--------------------------------------------------------*  svy: glm difunctional mpe i.sex i.age_group if survey == 1, fam(binomial) link(log) nolog eform  *--------------------------------------------------------*  * Slope Index of Inequality (SII) – single year  *--------------------------------------------------------*  svy: glm difunctional mpe i.sex i.age_group if survey == 1, am(binomial) link(identity)  *--------------------------------------------------------*  * RII with interaction (combined surveys)  *--------------------------------------------------------*  gen ridit_survey = ridit_score * survey  svy: glm difunctional ridit_score survey ridit_survey i.sex i.age_group, fam(binomial) link(log) nolog eform  *--------------------------------------------------------*  * SII with interaction (combined years)  *--------------------------------------------------------*  svy: glm difunctional mpe ano ridit_survey sexo i.age group  fam(binomial) link(identity)  *--------------------------------------------------------*  * Concentration Index with comparison across years  *--------------------------------------------------------*  conindex difunctional [aweight = wgt], ///  Ranvir(school cluster(single extract) ///  tresero robust loud compare(ano) |
| --- |

**Supplementary Results**

**Table S3:** Number and percentage of complete records for each analyzed variable by survey

| *Surveys* | *2003* | *2010* | *2023* | *Total sample* |
| --- | --- | --- | --- | --- |
| *Total sample* | 5,347 | 7,619 | 9,745 | 22,711 |
| ***Variables*** |  |  |  |  |
| *Brazilian regions* | 5,347 (100%) | 7,619 (100%) | 9,745 (100%) | 22,711 (100%) |
| *Sex* | 5,347 (100%) | 7,619 (100%) | 9,744 (99.99%) | 22,710 (99.99%) |
| *Age* | 5,347 (100%) | 7,619(100%) | 9,736 (99.91%) | 22,702 (99.96%) |
| *Education* | 5,262 (98.41%) | 7,421(97.40%) | 9,510 (97.59%) | 22,196 (97.73%) |
| *Edentulism* | 5,347 (100%) | 7,509 (98.56%) | 9,720 (99.74%) | 22,576 (99.41%) |
| *Functional dentition* | 5,347 (100%) | 7,509 (98.56%) | 9,720 (99.74%) | 22,576 (99.41%) |
| *All variables* | 5,262 (98.41%) | 7,316 (96.02%) | 9,502 (97.51%) | 22,080 (97.22%) |

Table S4: Prevalence of edentulism and functional dentition by education level among older adults in Brazil, 2003, 2010, and 2023, and differences in these prevalences between the surveys (**∆**2010 – 2003**,** ∆2023-2003, and ∆2023-2010), along with the Annual Percent Change (APC) over the study period.

| **Outcomes** | **2003 (n=5347)** | **2010 (n=7509)** | **2023 (n=9720)** | **∆2010 – 2003** | **% change** | ∆2023-2003 | **% change** | ∆2023-2010 | **% change** | APC (95% CI) |
| --- | --- | --- | --- | --- | --- | --- | --- | --- | --- | --- |
|  | **w% (95% CI)*** | **w% (95% CI)*** | **w% (95% CI)*** |  |  |  |  |  |  |  |
| Edentulism |  |  |  |  |  |  |  |  |  |  |
| **Brazil** | 53.34 (49.58; 57.09) | **53.38 (49.73; 57.03)** | **36.48 (33.37; 39.59)** | 0.04 (-5.19; 5.28) | 0.075 | **-16.86 (-21.74; -11.98)** | -31.61 | **-16.90 (-21.70; -12.11)** | -31.66 | **-2.01 (-3.44; -0,57)** |
| Did not study | 55.52 (51.97; 59.07) | **65.70 (58.23; 73.16)** | **54.11 (48.87; 59.36)** | **10.18 (1.91; 18.45)** | 18.33 | -1.41 (-7.75; 4.94) | -2.54 | **-11.58 (-20.71; -2.46)** | -17.64 | -0.30 (-2.24; 1.64) |
| 1 to 4 years of study | 58.69 (51.59; 65.78) | **57.74 (51.59; 65.78)** | **45.01 (39.53; 50.50)** | -0.95 (-9.58; 7.69) | -1.62 | **-13.67 (-22.64; -4.70)** | -23.31 | **-12.72 (-20.09; -5.36)** | -22.05 | **-1.39 (-2.22; -0.56)** |
| 5 to 8 years of study | 40.34 (32.65; 48.04) | **51.54 (46.14; 56.95)** | **38.03 (31.89; 44.16)** | **11.20 (1.80; 20.61**) | 27.76 | -2.32 (-12.16; 7.53) | -5.73 | **-13.52 (-21.69; -5.33)** | -26.21 | -0.55 (-3.45; 2.35) |
| 9 to 11 years of study | **41.64 (35.81; 47.46)** | 35.62 (27.46; 43.77) | **28.50 (22.99; 34.02)** | -6.02 (-16.04; 3.99) | -14.45 | **-13.13 (-21.15; -5.11)** | -31.56 | -7.11 (-16.95; 2.73) | -19.99 | **-1.85 (-2.11; -1.60)** |
| > 12 years of study | 21.81 (10.29; 33.32) | 21.23 (10.67; 31.80) | 13.27 (9.82; 16.72) | -0.57 (-16.20; 15.06) | -2.66 | -8.53 (-20.55; 3.49) | -39.16 | -7.96 (-19.07; 3.15) | -37.49 | **-2.59 (-4.16; -1.02)** |
| Functional dentition |  |  |  |  |  |  |  |  |  |  |
| **Brazil** | 9.89 (8.41; 11.37) | 11.45 (9.22; 13.68) | 23.95 (21.24; 26.65) | 1.56 (-1.12; 4.24) | 15.77 | **14.06 (10.97; 17.14)** | 142.16 | **12.50 (8.99; 16.00)** | 109.17 | **4.69 (2.81; 6.56)** |
| Did not study | 8.63 (6.31; 10.94) | 7.86 (1.67; 14.05) | 13.08 (9.38; 16.78) | -0.76 (-7.37; 5.84) | -8.92 | **4.45 (0.09; 8.82)** | 51.56 | 5.22 (-1.99; 12.43) | 66.41 | 2.34 (-0,34; 5.02) |
| 1 to 4 years of study | 8.47 (5.83; 11.12) | 9.44 (6.32; 12.55) | 16.79 (11.82; 21.75) | 0.97 (-3.12; 5.05) | 11.45 | **8.32 (2.69; 13.94)** | 98.23 | **7.35 (1.49; 13.21)** | 77.86 | **3.61 (2.2; 5.11)** |
| 5to 8 years of study | 10.65 (2.40; 18.91) | 9.67 (5.90; 13.44) | 19.05 (15.13; 22.97) | -0.98 (-10.06; 8.10) | -9.20 | 8.40 (-0.75; 17.54) | 78.87 | **9.38 (3.94; 14.82)** | 97.00 | 3.25 (-0.16; 6.65) |
| 9 to 11 years of study | 17.83 (11.49; 24.17) | 17.65 (11.89; 23.41) | 26.97 (20.58; 33.36) | -0.18 (-8.75; 8.39) | -1.01 | **9.14 (0.13; 18.15)** | 51.26 | **9.32 (0.71; 17.92)** | 52.80 | **2.24 (0.50; 3.98)** |
| > 12 years of study | 20.59 (13.46; 27.73) | 33.66 (24.48; 42.83) | 49.94 (44.26; 55.62) | **13.06 (1.44; 24.69)** | 63.48 | **29.35 (20.22; 38.47)** | 142.54 | **16.28 (5.49; 27.07)** | 48.37 | **4.34 (2.27; 6.42)** |

Note: The percentage change (%∆) in prevalence between years was calculated using the formula: %∆ = [(Prevalence in the later year – Prevalence in the earlier year) / Prevalence in the earlier year] × 100. For example, the change from 2003 to 2023 reflects the proportional difference relative to the baseline in 2003. Negative values indicate a decrease in prevalence over time, while positive values indicate an increase. The APC represents the average annual rate of change in prevalence of edentulism or functional dentition over time, where negative values indicate a decrease and positive values indicate an increase. ∆ and APC values in bold denote statistically significant differences (95% CIs not crossing zero; p < 0.05).

*Analyses accounted for the effects of sample design and weighting. w% = Weighted prevalence; 95% CI = Confidence Interval).

Considering subpopulations based on education level, a reduction in the prevalence of edentulism was observed, especially in 2023 compared to 2010, with the greatest variations occurring among older adults with up to 8 years of schooling. A significant decrease between 2023 and 2003 was observed for older adults with 9 to 11 years of schooling, but it was not significant when comparing 2023 with 2010. Older adults with 12 or more years of schooling had the lowest prevalence of edentulism in all years, although these prevalences were not significantly different across the surveys. The differences in functional dentition prevalence for subgroups by education level revealed a significant increase in 2023 compared to 2003 and 2010 for almost all education levels. The prevalence of functional dentition among those with no education was significantly higher in 2023 compared to 2003, and there was no difference comparing 2023 and 2010. For other groups, except for those with 5 to 8 years of schooling, a difference was observed starting from 2010, with a trend toward an increase in prevalence in 2023 compared to 2010. For participants with 12 or more years of schooling, the largest differences (in percentage points) in functional dentition prevalence were observed in the comparisons between 2023 and 2003, as well as between 2023 and 2010 (Table S3).
